# Protective effects and mechanisms of high-dose vitamin C on sepsis-associated cognitive impairment in rats

**DOI:** 10.1038/s41598-021-93861-x

**Published:** 2021-07-15

**Authors:** Ning Zhang, Wei Zhao, Zhen-Jie Hu, Sheng-Mei Ge, Yan Huo, Li-Xia Liu, Bu-Lang Gao

**Affiliations:** Department of Critical Care Medicine, The Fourth Hospital of Hebei Medical University, 12 Jiankang Road, Shijiazhuang, 050011 Hebei China

**Keywords:** Biochemistry, Drug discovery, Microbiology, Diseases, Medical research

## Abstract

Sepsis survivors present long-term cognitive deficits. The present study was to investigate the effect of early administration of high-dose vitamin C on cognitive function in septic rats and explore its possible cerebral protective mechanism. Rat sepsis models were established by cecal ligation and puncture (CLP). Ten days after surgery, the Morris water maze test was performed to evaluate the behavior and cognitive function. Histopathologic changes in the hippocampus were evaluated by nissl staining. The inflammatory cytokines, activities of antioxidant enzymes (superoxide dismutase or SOD) and oxidative products (malondialdehyde or MDA) in the serum and hippocampus were tested 24 h after surgery. The activity of matrix metalloproteinase-9 (MMP-9) and expressions of nuclear factor erythroid 2-related factor 2 (Nrf2) and heme oxygenase-1(HO-1) in the hippocampus were measured 24 h after surgery. Compared with the sham group in the Morris water maze test, the escape latency of sepsis rats was significantly (P = 0.001) prolonged in the navigation test, whereas the frequency to cross the platform and the time spent in the target quadrant were significantly (P = 0.003) reduced. High-dose vitamin C significantly decreased the escape latency (P = 0.01), but increased the time spent in the target quadrant (P = 0.04) and the frequency to cross the platform (P = 0.19). In the CLP+ saline group, the pyramidal neurons were reduced and distributed sparsely and disorderly, the levels of inflammatory cytokines of tumor necrosis factor (TNF)-α, interleukin (IL)-6, and IL-10 in the serum and hippocampus were significantly increased (P = 0.000), the blood brain barrier (BBB) permeability in the hippocampus was significantly (P = 0.000) increased, the activities of SOD in the serum and hippocampus were significantly (P = 0.000 and P = 0.03, respectively) diminished while the levels of MDA in the serum and hippocampus were significantly (P = 0.007) increased. High-dose vitamin C mitigated hippocampus histopathologic changes, reduced systemic inflammation and neuroinflammation, attenuated BBB disruption, inhibited oxidative stress in brain tissue, and up-regulated the expression of nuclear and total Nrf2 and HO-1. High-dose vitamin C significantly (P < 0.05) decreased the levels of tumor necrosis factor- (TNF)-α, interleukin-6 (IL-6), MDA in the serum and hippocampus, and the activity of MMP-9 in the hippocampus, but significantly (P < 0.05) increased the levels of SOD, the anti-inflammatory cytokine (IL-10) in the serum and hippocampus, and nuclear and total Nrf2, and HO-1 in the hippocampus. In conclusion, high-dose vitamin C can improve cognition impairment in septic rats, and the possible protective mechanism may be related to inhibition of inflammatory factors, alleviation of oxidative stress, and activation of the Nrf2/HO-1 pathway.

## Introduction

Sepsis is defined as life-threatening organ dysfunction caused by a dysregulated host response to infection^1^. Despite recent progress in the treatment of sepsis, it remains the most frequent cause of death in intensive care units (ICU)^2^. Septic survivors have long-term neurological sequelae, especially significant declines in the cognitive function. More than 50% of sepsis survivors suffer from long-term cognitive impairment, especially in general memory, attention, and executive function, consequently reducing their daily quality of life^3,4^. Sepsis potentially increases the brain susceptibility to neurodegenerative diseases and the risk of developing dementia in the future^5^.

Many studies have revealed the complex mechanisms of cognitive impairment in septic patients, involving neuroinflammatory response, oxidative stress, blood–brain-barrier (BBB) damage, mitochondrial dysfunction, and neuronal apoptosis in the brain tissue^6,7^. The Nrf2 (nuclear factor erythroid 2-related factor 2) signaling pathway protects the cell against oxidative stress and inflammation by upregulating the expression of numerous antioxidant genes in response to oxidative stress^8^.

Vitamin C or ascorbic acid is a water-soluble vitamin that acts as an enzyme cofactor and plays an important role in regulating oxidative stress and inflammatory response in many important biological reactions^9^. It plays a vital role in neuronal differentiation, maturation, and myelin formation^10^. Recently, it has been revealed that vitamin C also provides neuroprotective effects in many neurological diseases including Alzheimer’s disease and stroke^11,12^. Sepsis is associated with an acute deficiency of vitamin C^13^, and it has been proved that parenteral vitamin C alleviated organ injury and improved survival in septic rats^14^. Despite these benefits of vitamin C in experimental sepsis, studies concerning the effects of vitamin C treatment on cognition impairment associated with sepsis are still limited. It was hypothesized that high-dose vitamin C played an important role in protecting sepsis-associated cognitive impairment, and this study was consequently performed to investigate the effect of high-dose vitamin C on protecting sepsis-associated cognitive impairment in a rat sepsis model.

## Results

### Rat modeling

Six hours after modeling, the rats began to show signs and symptoms of hair erection, body curling, reduced activity, increased secretion of eyes, and dim hair color, indicating successful establishment of the sepsis model. Within 7 days after the establishment of the sepsis model, 9 rats died in the CLP (cecal ligation and puncture) + saline group with a survival rate of 47.1% (8/17), whereas 7 died in the CLP + vitamin C group resulting in a survival rate of 58.8% (10/17). Compared with CLP Group, rats in the vitamin C group woke up earlier, had better mental state, drank water earlier, and had more activities, but their hair color was dim, with increased secretion of both eyes. No significant (P = 0.32) difference existed in the survival rate between the CLP + saline and the CLP + Vitamin C groups. No rats died afterwards. All six rats were still alive in the sham group. The survived rats had the Morris water maze test.

### High-dose vitamin C improved cognitive impairment in sepsis rats

**Figure 1:**
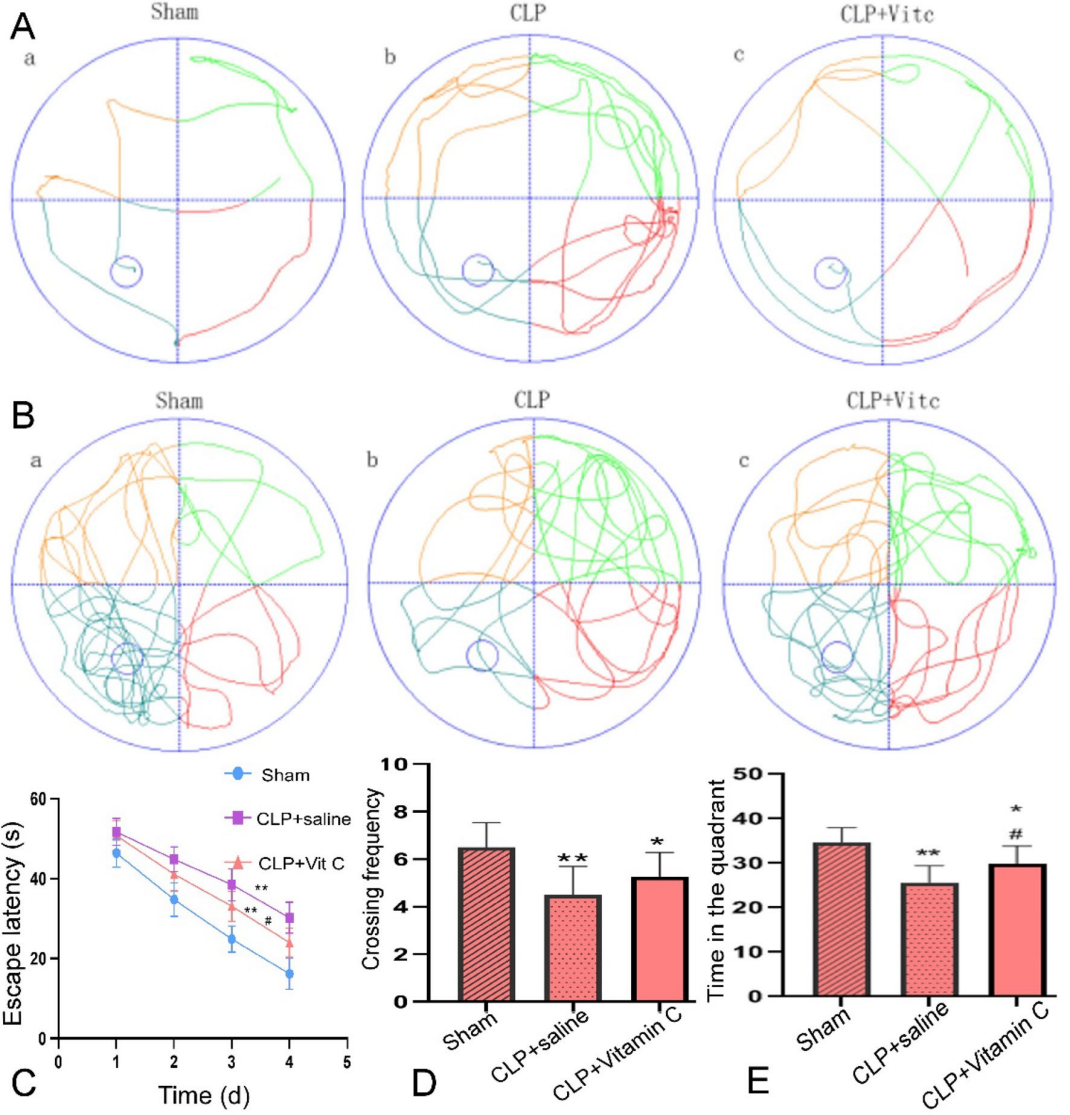
The performance of rats in three groups in the Morris water maze test. (**A**) Typical behavioral traces of three groups rats in the place navigation test. The more lines in the quadrants, the longer the escape latency. The rats in the CLP sepsis model group had significantly (P = 0.001) longer escape latency compared with the Sham and CLP + vitamin C groups. (**B**) Typical behavioral traces of the rats in three groups in the spatial probe test. The denser the blue lines in the quadrant where the platform is, the longer the retention time of rats. The rat retention time was significantly (P = 0.001) elongated in the platform quadrant in the Sham and CLP + vitamin C group compared with the CLP sepsis model group. (**C**) Comparison of the escape latency of the rats in three groups. The escape latency was significantly (P = 0.003) improved in the CLP + vitamin C group compared with the CLP model group. D&E. Comparison of the crossing frequency (**D**) and time (**E**) spent in the target quadrant of the rats in three groups, with significant improvement in the CLP + vitamin C group compared with CLP group. *P < 0.05 and **P < 0.01 compared with sham group; ^#^*P < *0.05 compared with CLP + saline group. *CLP* cecal ligation and puncture, *Vit C* vitamin C.

In the first 4 days of training in the Morris water maze test, the escape latency of rats in each group was gradually shortened with training as the days passed (P = 0.000). Compared with the sham group (Fig. 1), the escape latency of sepsis rats was significantly (P = 0.001) prolonged in the navigation test, whereas the frequency to cross the platform and the time spent in the target quadrant were significantly (P = 0.003) reduced. Compared with the sepsis rats with saline injection only (CLP + saline group), vitamin C significantly decreased the escape latency (P = 0.01), but increased the time spent in the target quadrant (P = 0.04) and the frequency to cross the platform (P = 0.19).

### High-dose vitamin C mitigated hippocampal histopathologic changes

**Figure 2:**
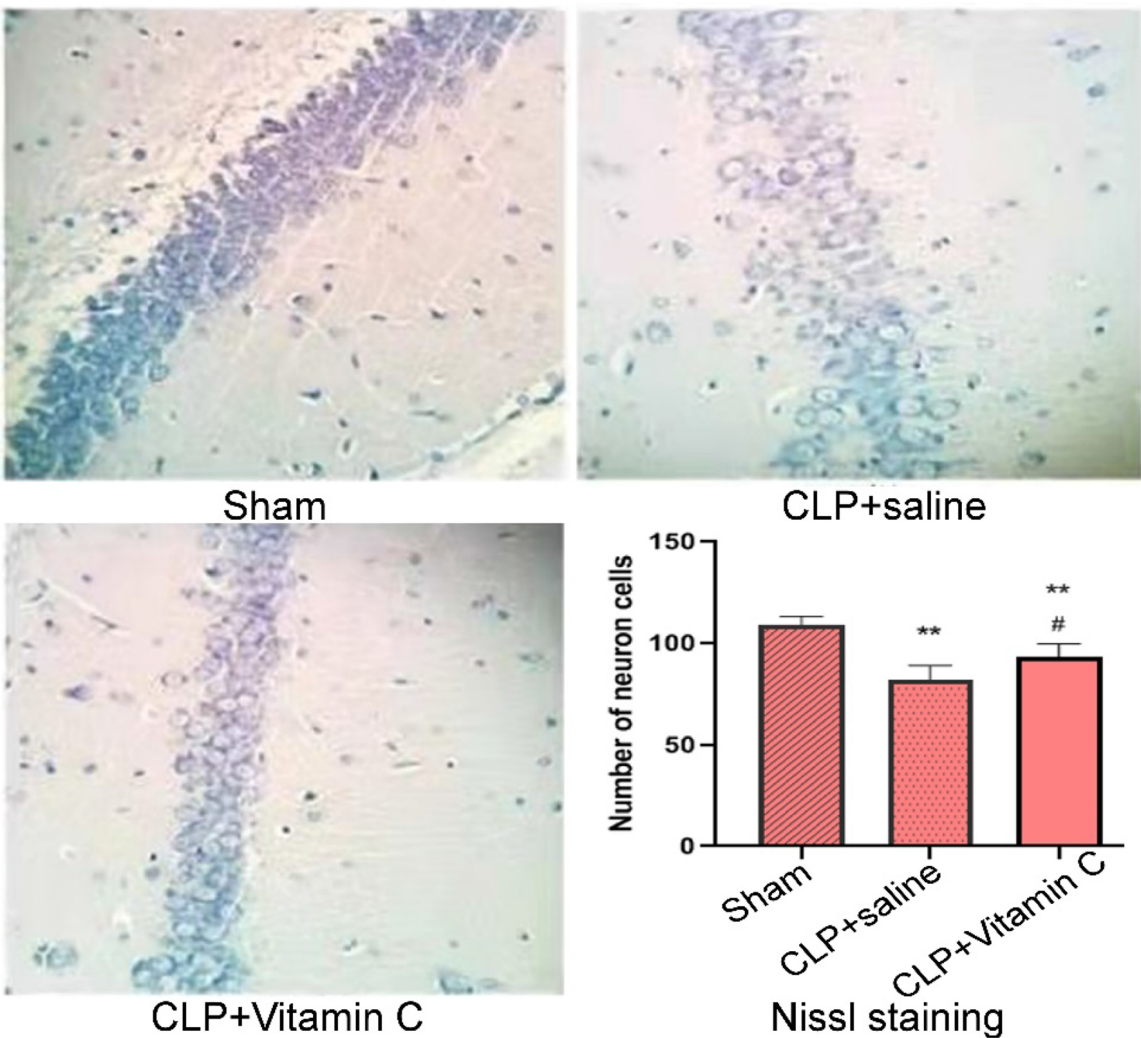
Nissl staining in the hippocampal CA1 region. Representative sections from the hippocampal CA1 region were observed 24 h after operation (Nissl staining × 200). The pyramidal neurons in the hippocampal CA1 region were significantly decreased (P = 0.000) in both the CLP + saline group and the CLP + vitamin C group compared with the sham group. Compared with the CLP + high-dose vitamin C group, the pyramidal neurons in the CLP + saline group were significantly (P = 0.01) decreased. *P < 0.05 and **P < 0.01 compared with sham group; ^#^*P < *0.05 compared with the CLP group. CLP, cecal ligation and puncture. Statistical value: 24.69, total degree of freedom: 14, P: 0.000.

In the sham group (Fig. 2), the pyramidal neurons in the hippocampal CA1 region were clearly discernible and neatly arranged, with abundant Nissl bodies in the cytoplasm. In the CLP + saline group, the pyramidal neurons were distributed sparsely and disorderly, with reduced and dissolved Nissl bodies. Compared with the sham group, the number of pyramidal neurons in the CLP group significantly decreased (P = 0.000). In the CLP + high-dose vitamin C group, the pyramidal neurons were better arranged, and the number of the pyramidal neurons was significantly (P = 0.01) greater than that in the CLP + saline group, suggesting that vitamin C had relieved the hippocampal pathological damage in septic rats.

### High-dose vitamin C reduced systemic inflammation and neuroinflammation

**Figure 3:**
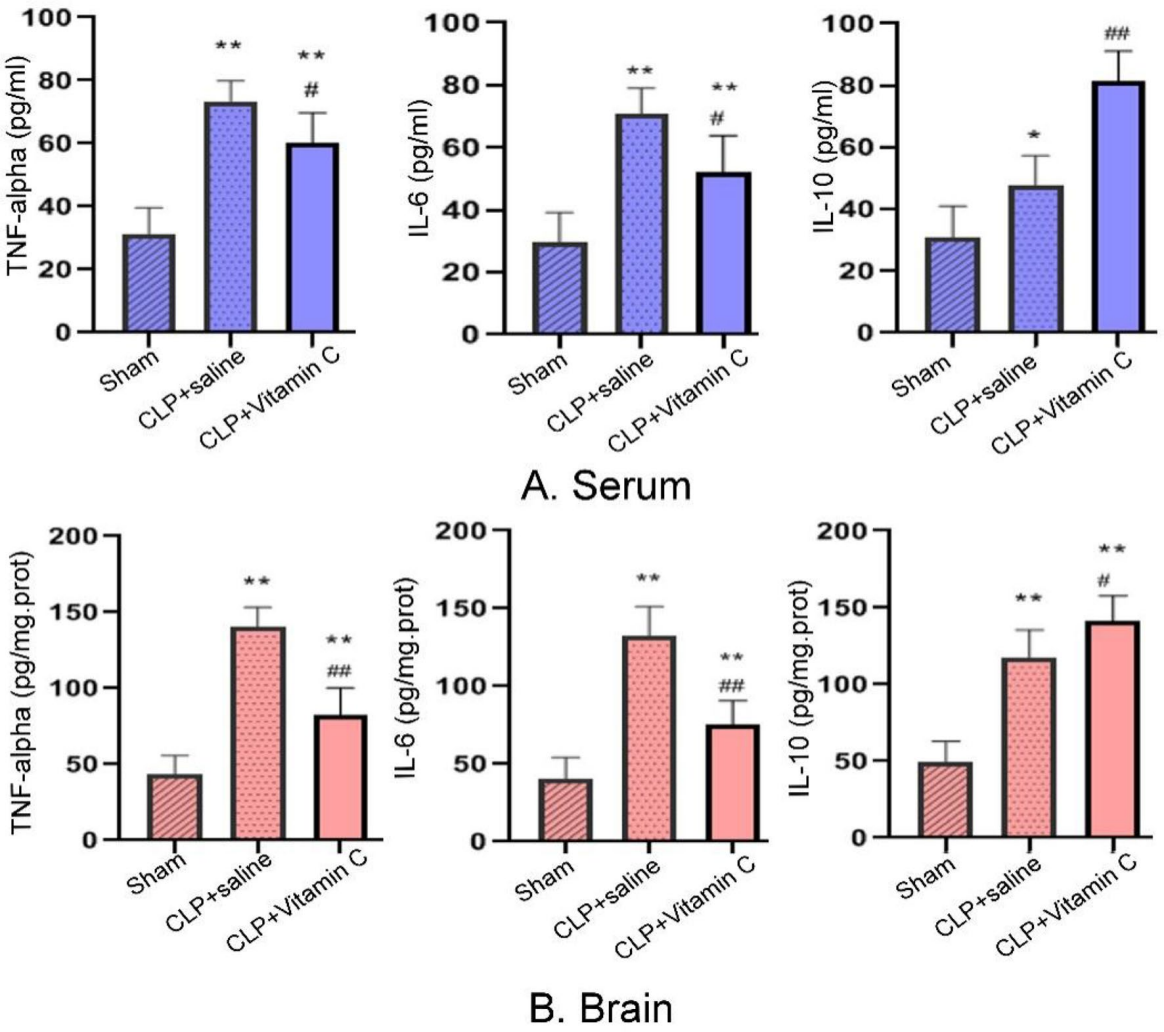
Comparison of TNF-α, IL-6 and IL-10 levels in the serum (**A**) and brain hippocampus (**B**) of rats in three groups 24 h after operation. Compared with the sham group, the levels of inflammatory cytokines of TNF-α, IL-6, and IL-10 in the serum and hippocampus in both the CLP + saline group and CLP + vitamin C group were significantly increased (P = 0.000). Compared with the CLP + saline group, the levels of TNF-α and IL-6 in the serum and hippocampus in the CLP + vitamin C group were significantly decreased (P = 0.001). The anti-inflammatory cytokine (IL-10) in the serum and hippocampus were significantly increased (P = 0.001).*P < 0.05 and **P < 0.01 compared with sham group; ^#^*P < *0.05 and ^##^*P < *0.01 compared with CLP group. *TNF-α* tumor necrosis factor, *IL-6* interleukin-6, *IL-10* interleukin-10, *CLP* cecal ligation and puncture.

The inflammatory cytokines were detected in the serum and hippocampus of rats 24 h after the sepsis modeling surgery (Fig. 3). The cytokines range measured in the serum was 0–100 pg/ml, and the cytokines range measured in the brain hippocampal tissue was 0–200 pg/mg prot. Compared with the sham group, the levels of inflammatory cytokines of tumor necrosis factor (TNF)-α, interleukin (IL)-6, and IL-10 in the serum and hippocampus in both the CLP + saline group and CLP + vitamin C group were significantly increased (P = 0.000). Compared with the CLP + saline group, the levels of TNF-α and IL-6 in the serum and hippocampus in the CLP + vitamin C group were significantly decreased (P = 0.001). The anti-inflammatory cytokine (IL-10) in the serum and hippocampus were significantly increased (P = 0.001). There were positive correlations between the serum and hippocampus concentrations of TNF-α, IL-6, and IL-10 (P = 0.000, Pearson correlation = 0.871; P = 0.000, Pearson correlation = 0.874; P = 0.001, Pearson correlation = 0.768, respectively).

### High-dose vitamin C attenuated BBB disruption

**Figure 4:**
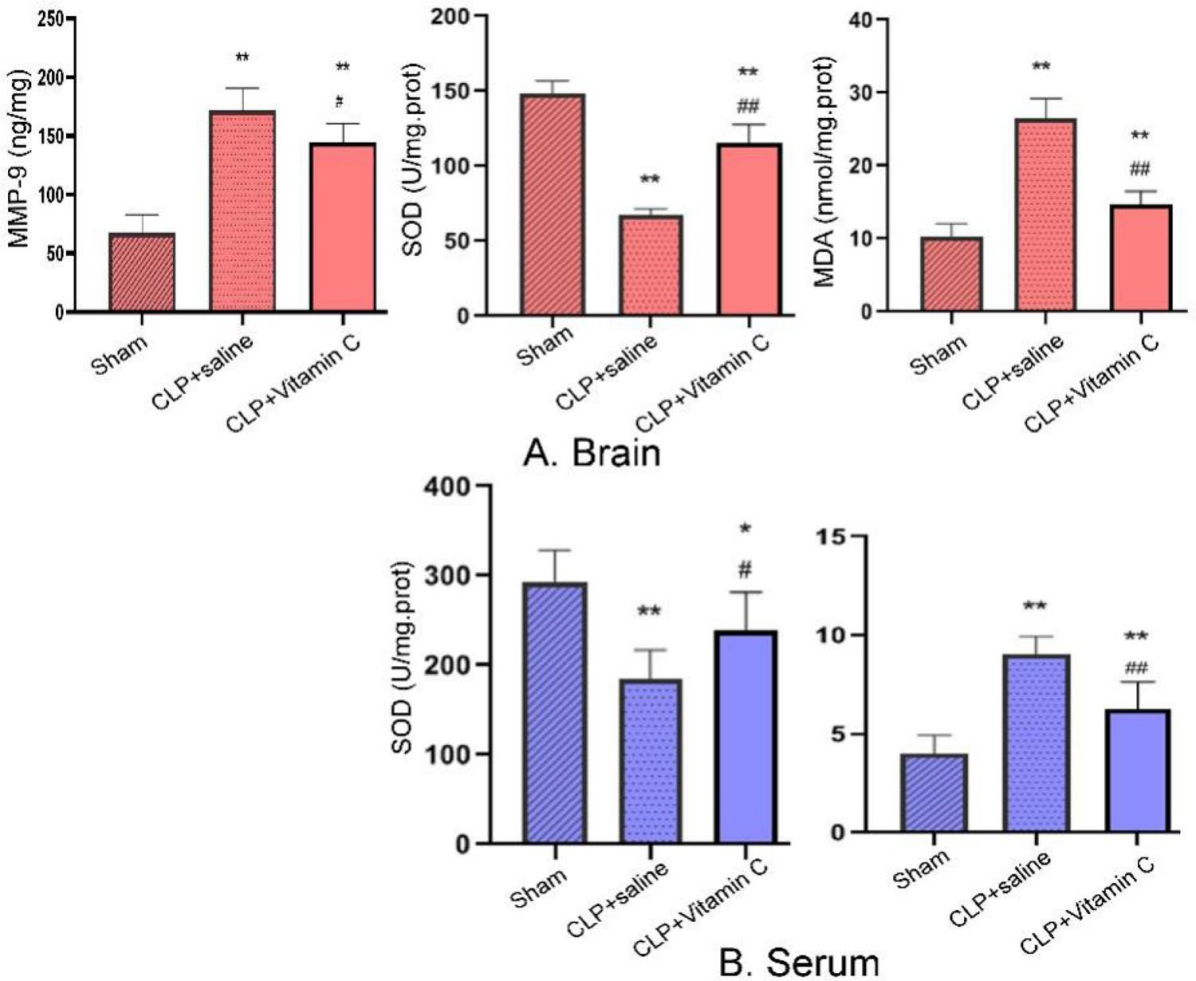
Comparison of the MMP-9 (24 h after operation), SOD and MDA levels in brain hippocampus and serum in three groups. MMP-9 in the hippocampus was significantly (P = 0.000) increased in the hippocampus in both the CLP + saline and the CLP + vitamin C groups, but significantly (P = 0.02) reduced with treatment of vitamin C compared with the CLP + saline group. Compared with the sham group, the activities of SOD in the serum and hippocampus were significantly (P = 0.000 and P = 0.03, respectively) diminished while the levels of MDA in the serum and hippocampus were significantly (P = 0.007) increased in both the CLP + saline group and the CLP + vitamin C group. Compared with the CLP + saline group, the levels of SOD were significantly (P = 0.03 and P = 0.000, respectively) increased while the levels of MDA were significantly (P = 0.002 and P = 0.000, respectively) decreased in the serum and hippocampus in the CLP + vitamin C group. *P < 0.05 and **P < 0.01 compared with sham group; ^#^*P < *0.05 and ^##^*P < *0.01 compared with CLP group. *MMP* matrix metalloproteinase, *SOD* superoxide dismutase, *MDA* malondialdehyde, *CLP* cecal ligation and puncture.

The BBB permeability measured by the activity of matrix metalloproteinase-9 (MMP-9) in the hippocampus was significantly (P = 0.000) increased in the hippocampus in both the CLP + saline and the CLP + vitamin C groups, but significantly (P = 0.02) reduced with treatment of vitamin C compared with the CLP + saline group (Fig. 4).

### High-dose vitamin C inhibited oxidative stress in brain tissue

The antioxidant enzyme SOD and oxidative product MDA were detected in the serum and hippocampus in each group 24 h after the modeling surgery. Compared with the sham group, the activities of superoxide dismutase (SOD) in the serum and hippocampus were significantly (P = 0.000 and P = 0.03, respectively) diminished while the levels of malondialdehyde (MDA) in the serum and hippocampus were significantly (P = 0.007) increased in both the CLP + saline group and the CLP + Vitamin C group. Compared with the CLP + saline group, the levels of SOD were significantly (P = 0.03 and P = 0.000, respectively) increased while the levels of MDA were significantly (P = 0.002 and P = 0.000, respectively) decreased in the serum and hippocampus in the CLP + Vitamin C group.

### High-dose vitamin C up-regulated expression of nuclear and total Nrf2 and HO-1

**Figure 5:**
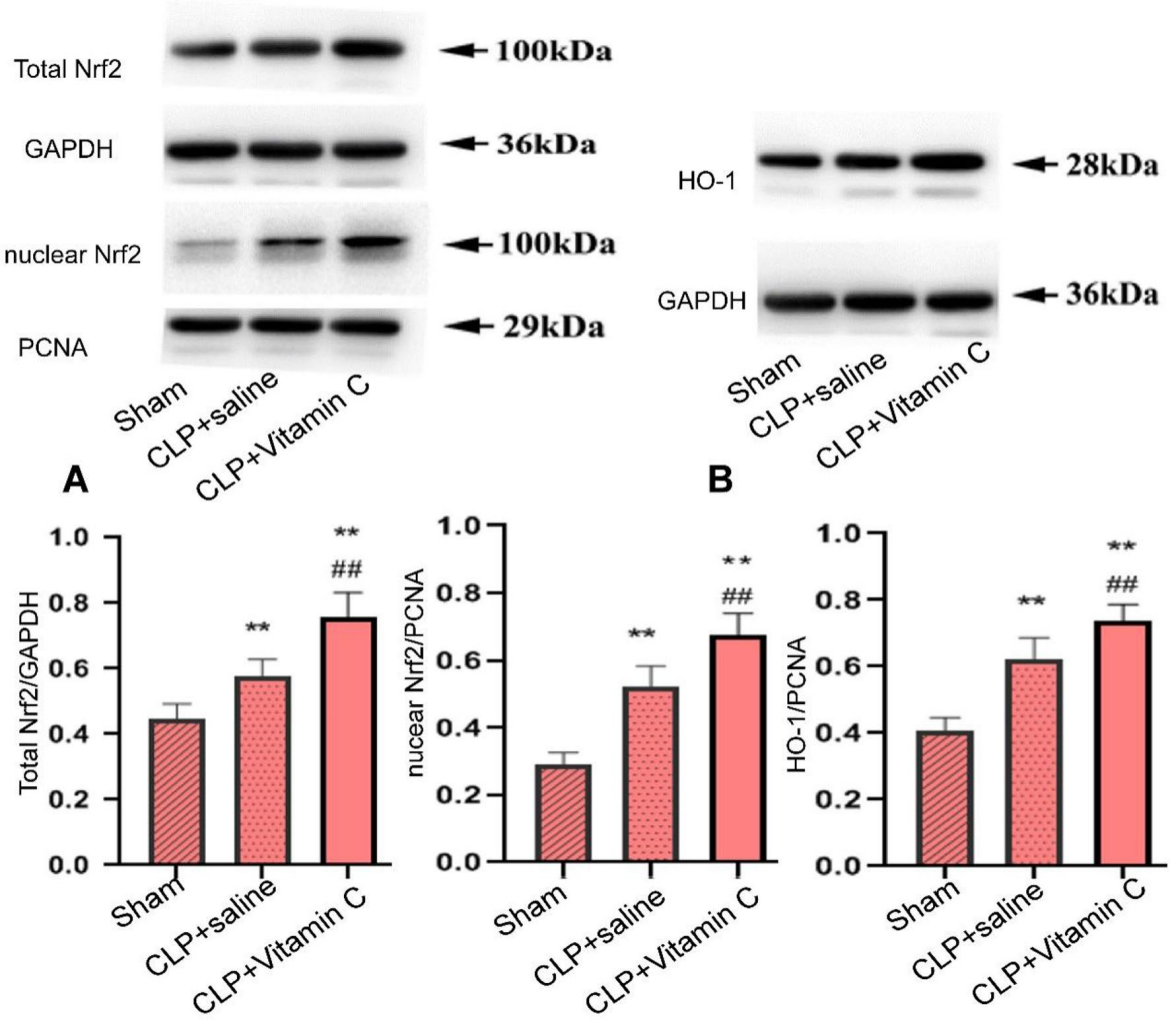
Comparison of the protein expression levels of the total and nucleus Nrf2 and cytoplasmic HO-1 in three groups of CLP-induced septic rats. (**A**) The expression of total and nuclear Nrf2 in hippocampus. (**B**) The expression of cytoplasmic HO-1 in hippocampus. Compared with the sham group, the nuclear and total Nrf2 and HO-1were significantly (P = 0.004) increased in both the CLP + saline group and the CLP + vitamin C group 24 h after CLP modeling surgery. Compared with the CLP + saline group, the expression levels of the nuclear and total Nrf2, and HO-1 were significantly (P = 0.001) increased in the CLP + Vitamin C group. Values are expressed as mean ± standard deviation (n = 5). *P < 0.05 and **P < 0.01 compared with the sham group; ^##^*P < *0.01 compared with CLP + saline group. *CLP* cecal ligation and puncture, *Nrf2* nuclear factor erythroid 2-related factor 2, *HO-1* heme oxygenase-1.

Compared with the sham group, the expression levels of the nuclear and total Nrf2 and HO-1 (heme oxygenase-1) were significantly (P = 0.000, P = 0.000, P = 0.004, and P = 0.000, respectively) increased in both the CLP + saline group and the CLP + vitamin C group 24 h after CLP modeling surgery (Fig. 5). Compared with the CLP + saline group, the expression levels of the nuclear and total Nrf2, and HO-1 were significantly (P = 0.001) increased in the CLP + vitamin C group.

## Discussion

In this study, the neuroprotective effect of vitamin C was investigated in a well-established rat model of experimental sepsis. It was found that the high-dose vitamin C can effectively alleviate neuroinflammation, oxidative stress damage, pathological damage, and cognitive impairment in rat sepsis models, and the Nrf2/HO-1 pathway activation by vitamin C treatment in the hippocampus of the septic rats may be the potential mechanism.

Septic survivors frequently present long-term cognitive impairment, leading to decreased quality of life^15^. In pre-clinical sepsis models, cognitive impairment, especially aversive memory and learning, has been identified from early hours after sepsis until several months after recovery^16^. With reference to previous research^17^, a behavioral test was performed 10 days after surgery. The aim of our experiment is to reduce the inflammation and oxidative stress, improve the permeability of blood–brain barrier and improve the cognitive function in the late stage of sepsis by early application of vitamin C. In our study, the Morris Water Maze test was conducted to evaluate the cognition function of rats. The Morris Water Maze test, a classic test for detecting spatial learning and memory, has become a widely used method for assessing cognitive function in animal models^18^ and is closely related to synaptic plasticity in the hippocampus^19^. In our study, rats in the CLP + saline group presented spatial learning and recognition memory deficits, which were improved by high-dose vitamin C treatment in the CLP + vitamin C group. The results are consistent with the pathological changes of neurons in the hippocampal CA1 region. Unfortunately, there was no significant (P > 0.05) statistical difference in the frequency to cross the platform among different rat groups.

It is well known that systemic inflammatory response plays an important role in the development of long-term cognitive impairment^20,21^. In sepsis, neuroinflammation induces microglia activation, resulting in neurons injury and apoptosis^22^. The hippocampus, as a temporal lobe structure associated with learning and memory, is vulnerable to systemic pro-inflammatory cytokines because of highly expressed pro-inflammatory cytokine receptors, for example, the relative prevalence of the TNF-α receptor^23,24^. In a previous study, inflammatory cytokines of sepsis survivors are associated with decreased learning and memory in the central nervous system^16^. In the present study, it was found that high-dose vitamin C administration had greatly decreased the levels of pro-inflammatory cytokine (TNF-α and Il-6), but increased the levels of anti-inflammatory cytokines (IL-10) in the hippocampus and serum of the septic rats, which may be associated with the improvement of cognition.

The BBB is a highly selective biological interface between the brain and the periphery, and numerous studies had found that BBB plays an important role in the progression of acute and chronic brain dysfunction^7^. Experimental studies suggest that increased levels of TNF-α in the serum activate MMP-9 in the brain, leading to increased BBB permeability^25^. In our study, it was found that BBB permeability was increased 24 h after CLP modeling surgery but was significantly improved by high-dose vitamin C.

During sepsis, brain disorder is associated with increased production of reactive oxygen species and reactive nitrogen species, decreased antioxidant capacity, and increased oxidative stress^26^. Reactive oxygen species and reactive nitrogen species have been shown to play an important role in the pathophysiology of sepsis, and excessive reactive oxygen species induce lipid peroxidation, leading to cell and mitochondrial membrane damage^27^. Free radical-mediated lipid peroxidation causes structural membrane damage in the brain parenchyma from the very early stage of sepsis^28^. The brain is often vulnerable to oxidative stress because of its high oxygen consumption and low antioxidant capacity during sepsis^29^. Antioxidant compounds usually protect the cells from oxidative damage by scavenging reactive oxygen species and reactive nitrogen species, thus reducing neurological conditions related to oxidative stress^30^. Experimental studies suggest that antioxidant treatment with *N*-acetylcysteine and deferoxamine prevents cognitive impairment in septic mice^31^. SOD can transform superoxide radicals into hydrogen peroxide and prevent attack of reactive oxygen species to vital organs. MDA is a credible indicator of reactive oxygen species-induced lipid peroxidation, and its level mediates the severity of the impairment induced by reactive oxygen species^32^. In our study, numerous impaired neurons were found in the hippocampal CA1 region of septic rats. High-dose vitamin C treatment alleviated neuron injury in sepsis, associated with increased activities of antioxidant but decreased levels of oxidative products in the brain. Therefore, reversing oxidative stress in the hippocampus may be the possible mechanism for vitamin C treatment to alleviate brain injury in sepsis.

The primary mechanism for maintenance of cellular redox balance and to reestablish homeostasis is through the Nrf2 pathway, in response to inflammation and oxidative stress. Keap1 is a cytoplasmic Nrf2-interacting protein that negatively regulates Nrf2 activity^33^. Under cellular stress, reactive oxygen species or oxidative/electrophilic molecules modify cysteine residues of Keap1. These modifications lead to conformational changes of Keap1 to release Nrf2, thus activating Nrf2 to initiate the transcription of many genes involved in the antioxidant and antiinflammatory responses^34^. Nrf2 activation alleviates the neurodegenerative disorders in the brain through upregulation of antioxidant defenses, decrease of inflammation response, improvement of mitochondrial function, and maintenance of protein homeostasis^8^. Our study showed that high-dose vitamin C treatment dramatically up-regulated both nuclear and total Nrf2 expression in the hippocampus, increased the concentration of antioxidant enzyme SOD, and up-regulated HO-1 protein expression to inhibit the inflammatory response. Thus, targeting Nrf2/HO-1 signaling may provide a therapeutic option to improve cognition impairment of septic rats.

In our study, we first tested the inflammatory factors, oxidative stress of Serum and hippocampus, BBB permeability, Nissl's staining of hippocampus, and Western blot of hippocampus 24 h after the establishment of the CLP-induced sepsis model, and the behavioral test was performed 10 days after establishment of the CLP sepsis model. The study by Brichellod et al. pointed out that the rats had aversive learning and memory impairment 10 days after the establishment of CLP-induced sepsis model^35^, which was confirmed by our study. All the other experiments were performed 24 h after the model establishment because early application of vitamin C in sepsis rats can improve the decline of learning and memory ability in late sepsis. The results of biochemical parameters measured 24 h after model establishment showed that early application of vitamin C could improve the ability of learning and memory decrease in late sepsis, which is related to the improvement of biochemical parameters of sepsis.

Some limitations may exist in our study. Firstly, the number of rats used in this study was small, and more rats are needed for better outcomes. In this study, only high doses of vitamin C were used, and different dosages of vitamin C should be used to find the best dosage for improving the cognitive impairment. Moreover, this study did not investigate the mechanism of high-dose vitamin C in improving the cognitive function in septic rats from the perspective of the Nrf2 and HO-1 gene levels. Future studies will have to address these issues for better outcomes.

In conclusion, high-dose vitamin C treatment alleviates cognitive dysfunction and pathological damages in the brain hippocampus in CLP-induced septic rats. The neuroprotective effects of high-dose vitamin C on cognitive impairment are associated with decreases in neuroinflammation, oxidative stress, and BBB permeability through activating the Nrf2/HO-1 signaling pathway.

## Methods

### Animals

Sixty-one adult male SD rats, aged 6–8 weeks and weighed 200–250 g, were purchased from the Huafukang Biotechnology Co, Ltd (Beijing, China) and used in this study. All the rats were housed in cages with free access to food and water and were maintained on a 12-h light/dark cycle (lights on at 7 a.m.). The rats were randomly divided into three groups: (1) sham + saline, (2) CLP + saline, and (3) CLP + high-dose vitamin C (200 mg/kg via intraperitoneal injection)^36^, with 11 rats in the sham group and 25 in each of the CLP + saline group and CLP + high-dose vitamin C group. This study and all experimental procedures were approved by the Ethics Committee of the Fourth Hospital of Hebei Medical University (IACUC-4th hosHebmu) and were carried out in compliance with the ARRIVE guidelines. All methods were performed in accordance with the relevant guidelines and regulations.

### CLP model and treatment

Sepsis was induced by CLP as previously described^37^. Briefly, rats were anesthetized (general anesthesia) intraperitoneally (i.p.) with a mixture of ketamine (80 mg/kg) and xylazine (10 mg/kg). Under aseptic conditions, a 3-cm midline laparotomy was performed to expose the cecum and adjoining intestine. The cecum was tightly ligated with a 4.0 silk suture at 1/3 of the entire proximal cecum below the ileocecal valve before being perforated twice with an 18-gauge needle and squeezed gently to extrude a small amount of feces through the perforation site. The cecum was then returned to the peritoneal cavity, and the laparotomy was closed with 4.0 silk sutures. Animals were resuscitated with regular saline (30 mL/kg) subcutaneously (s.c.) immediately after CLP and imipenem sistatin sodium (14 mg/kg) s.c. immediately after and 12 h after CLP. Thirty minutes and 12 h after the CLP surgery, rats were treated with peritoneal injection of high-dose vitamin C 200 mg/kg^14^ in the CLP + high-dose vitamin C group or the same amount of saline in the CLP + saline group. This dose was used because it could improve multiple organ dysfunction in sepsis^14^. Vitamin C was obtained from the solarbio Biotechnology Co, Ltd (Beijing, China) and dissolved in saline before use. In the sham group, rats were subjected to all surgical procedures, but without ligation or perforation of the cecum. To minimize variability between different experiments, the same investigator performed all the CLP procedures.

Twenty-four hours after the CLP modeling surgery, rats (5 in the sham + saline and 8 in each of the CLP + saline group and the CLP + high-dose vitamin C group) for biochemical evaluation and brain tissue testing were subjected to a painless death with thiopental overdose (0.5 g/kg, i.p.) followed by decapitation. The brain structure hippocampus was quickly isolated, resected, and stored at − 80 °C for further analysis. Behavioral tests were performed 10 days after CLP modeling surgery in the rest rats, with six in the sham group and 17 in each of the CLP + saline and CLP + high-dose vitamin C groups.

### Morris water maze test

The Morris water maze test, including the space navigation test for 4 days and spatial probe test for 1 day, was performed to evaluate the rat functions in spatial learning and memory. The apparatus was a black circular pool of 150 cm in diameter and 60 cm high and was filled with warm (20 ± 1 °C) water, with the water temperature kept at 20 ± 1 °C during the whole experiment. During the experiment, an object of reference was set up at a fixed location outside the water maze. The escape platform was 9 cm in diameter and 1.0 cm below the water surface. The space navigation test lasted 4 consecutive days, with four trials per day. If the rat failed to find the platform within 90 s, the latency time was recorded as 90 s, and the rat was gently guided to the platform by the experimenter. After the space navigation test, the spatial probe test was performed on the fifth day, and the rats were allowed to swim for 90 s. The time spent in the target quadrant and the frequency to cross the platform were recorded. The behavioral test was recorded by the same person who was blind to the rat group. The data for the behavioral tests were collected and analyzed using the JLBehv behavioral analysis software (Ji Liang Science and Technology Company Limited, Shanghai, China).

### Cytokine measurements

The concentrations of TNF-α, IL-6, and IL-10 in the serum and the hippocampal tissue were assayed with a commercial enzyme-linked immunosorbent assay kit (EIAAB Science, Wuhan, China). All the procedures were performed according to the manufacturer’s protocols.

### Permeability of BBB

The concentration of MMP-9 in the brain tissue was used to evaluate the BBB permeability^38^. The level of MMP-9 in the hippocampus was measured by using commercially available ELISA kits (EIAAB Science, Wuhan, China).

### Measurement of SOD and MDA

According to the manufacturer’s instructions, the hippocampus was collected to measure the activity of MDA and SOD using commercially available kits (Jiancheng Biotechnology, Nanjing, China). The tissue homogenate of the hippocampus of 10% was prepared for biochemical assays. After addition of 9 times volume of pre-cooled PBS (mL) containing RMSF and RIPA cell lysate to the hippocampeal tissue homogenate, centrifugation was carried out at 4 °C and 15,000 rpm for 15 min. Then, the levels of SOD activity and MDA contents in the hippocampal tissues were determined by spectrophotometry and measured by means of microplate reader, respectively. All the procedures were performed according to the manufacturer protocols.

### Western blotting analysis

The expression of Nrf2 nucleoprotein, total protein, and HO-1 in the hippocampal tissue was assayed with the Western blotting technique^8^. The hippocampus tissues were obtained 24 h after operation and stored at – 80 °C. Protein extraction was performed by following the instructions of the total protein and nuclear protein extraction kit (Thermo, USA), and protein quantification was conducted with a BCA Protein Assay kit (Solaibo, China). The protein samples were divided in equal amounts using the sodium dodecyl sulfatepolyacrylamide gel electrophoresis (SDS-PAGE) and transferred to a nitrocellulose membrane. After 30 μg protein was added to the gel, the membranes were blocked with 5% skim milk TSB buffer for 2 h before being incubated with primary antibodies (Nrf2, 1:1000; GAPDH, 1:5000; HO-1, 1:1000; PCNA, 1:5000, Sanying, Wuhan, China) overnight at 4 °C. After five washes with TBST, the membranes were incubated with goat anti-mouse secondary antibody (1:10,000, Sigma, USA) at room temperature for 2 h. The enhanced chemiluminesence (Bio-Rad, USA) was used to observe the blots following the manufacturer’s instruction, and the quantification of the band relative density was measured using the densitometry (Molecular Analyst Image-analysis Software, Bio-Rad, USA).

### Nissl staining

**Figure 6:**
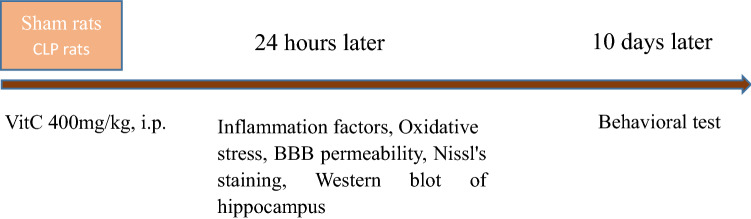
Experimental design. Twenty-four hours after CLP-induced sepsis model was established, inflammatory factors, oxidative stress of Serum and hippocampus, BBB permeability, Nissl's staining of hippocampus, and Western blot of hippocampus were evaluated. Ten days after CLP-induced sepsis model, behavioral tests of rats were performed.

After the rats were deeply anesthetized, the brains were harvested to evaluate the hippocampal damage 24 h after the model surgery. After transcardial perfusion with 4% paraformaldehyde in PBS, the hippocampal tissue was obtained, fixed with 10% formalin for 24 h, and then embedded in paraffin. Coronal sections at 5-mm thickness were taken from the Nissl body using the cresyl violet after deparaffinization and rehydration. All experiments and experiment design were shown in Figs. 1, 2, 3, 4, 5, 6.

### Statistical analysis

All statistical analyses were performed using the SPSS software 23.0 (IBM, Chicago, IL, USA). All data were presented as mean ± S.D (standard deviation). Comparison of multiple groups was performed by one-way analysis of variance (ANOVA), and intergroup comparison was conducted by the LSD test. The Escape latency in the Morris water maze test was analyzed using ANOVA. P < 0.05 was considered statistically significant.

## Supplementary Information

Supplementary Information.

## Data Availability

Anonymized data and analytic methods that support the findings of this study are available from the principal investigator on reasonable request.
