# Author Correction: Protective effects and mechanisms of high-dose vitamin C on sepsis-associated cognitive impairment in rats

**DOI:** 10.1038/s41598-022-05819-2

**Published:** 2022-01-28

**Authors:** Ning Zhang, Wei Zhao, Zhen-Jie Hu, Sheng-Mei Ge, Yan Huo, Li-Xia Liu, Bu-Lang Gao

**Affiliations:** Department of Critical Care Medicine, The Fourth Hospital of Hebei Medical University, 12 Jiankang Road, Shijiazhuang, 050011 Hebei China

Correction to: *Scientific Reports* 10.1038/s41598-021-93861-x, published online 15 July 2021

The original version of this Article contained an error in Figure 6, as the method of VitC administration was incorrectly described as a subcutaneous injection, rather than intraperitoneal.

The original Figure 6 and accompanying legend appear below.

**Figure 6:**
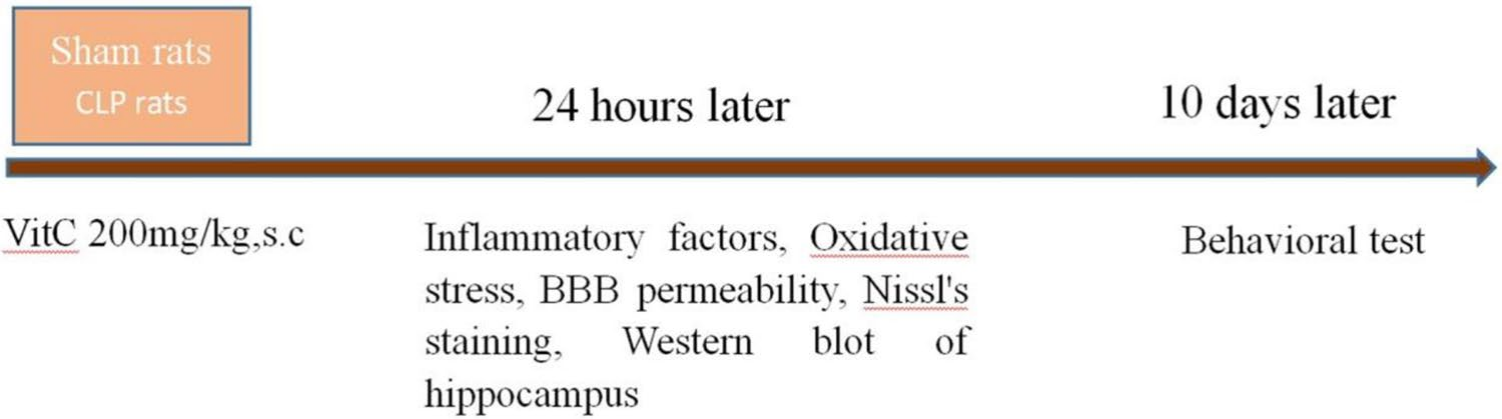
Experimental design. Twenty-four hours after CLP-induced sepsis model was established, inflammatory factors, oxidative stress of serum and hippocampus, BBB permeability, Nissl’s staining of hippocampus, and Western blot of hippocampus were evaluated. Ten days after CLP-induced sepsis model, behavioral tests of rats were performed.

The original Article has been corrected.

