# Supplementary material for: Protective effects and mechanisms of high-dose vitamin C on sepsis-associated cognitive impairment in rats
Source: Sci Rep. 2021 Jul 15;11:14511. doi: 10.1038/s41598-021-93861-x (PMC8282649; doi:10.1038/s41598-021-93861-x)
Supplement: Supplementary file 1 — Supplementary Information. [file 41598_2021_93861_MOESM1_ESM.pdf]

Supplemental figures of the original strips in Fig.5 A and B.

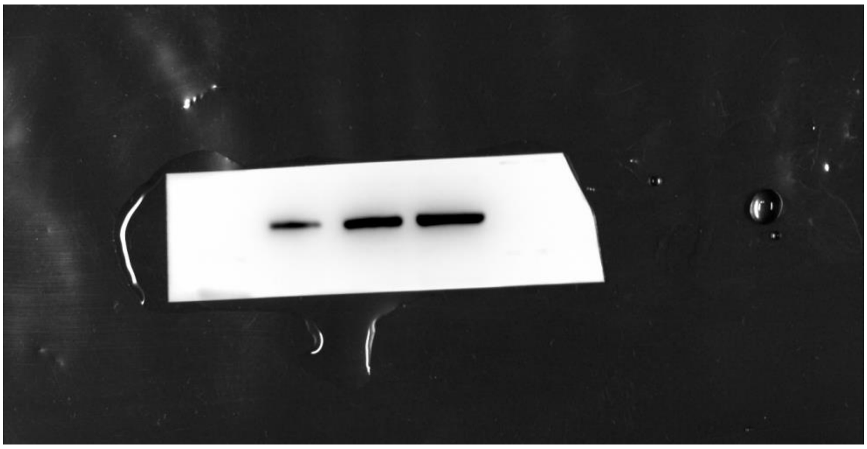

Original strip of Total Nrf2 in Fig.A

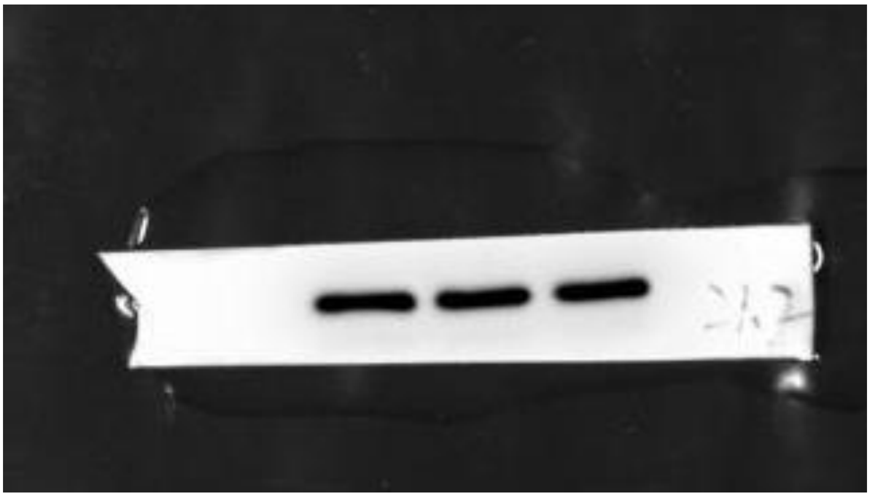

Original strip of GAPDH in Fig.A

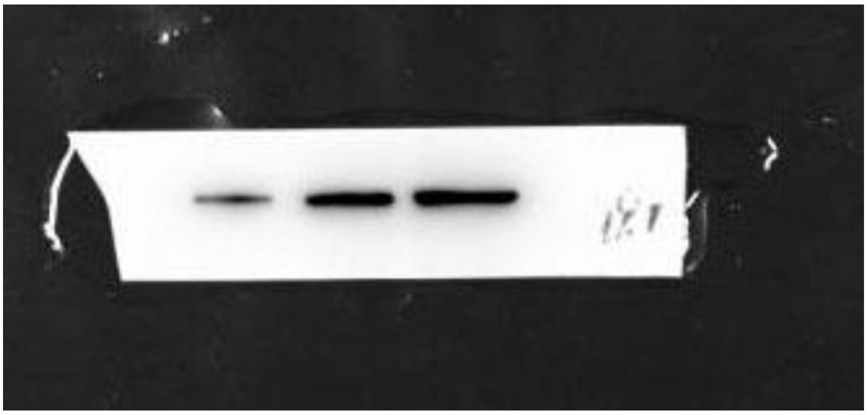

Original strip of nuclear Nrf2 in Fig.A

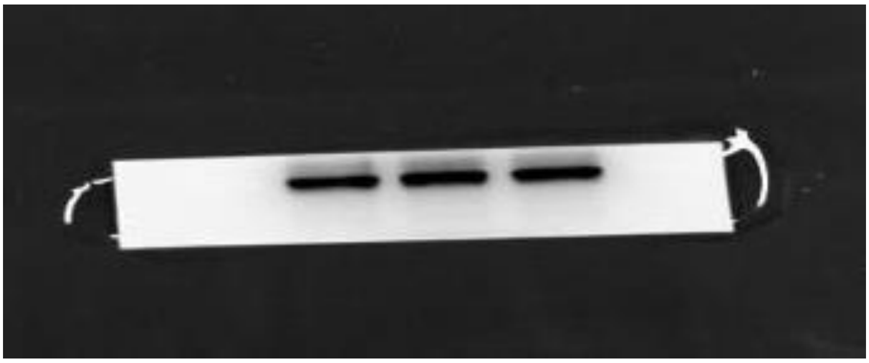

Original strip of PCNA in Fig.A

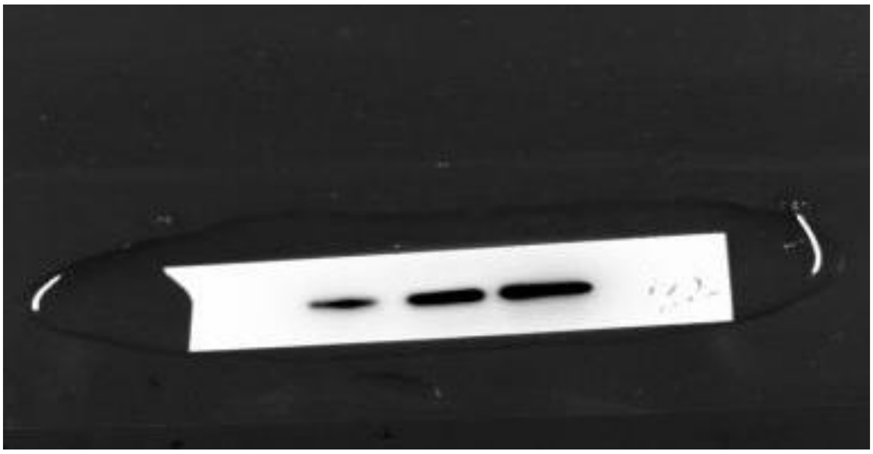

Original strip of HO-1 in Fig.B

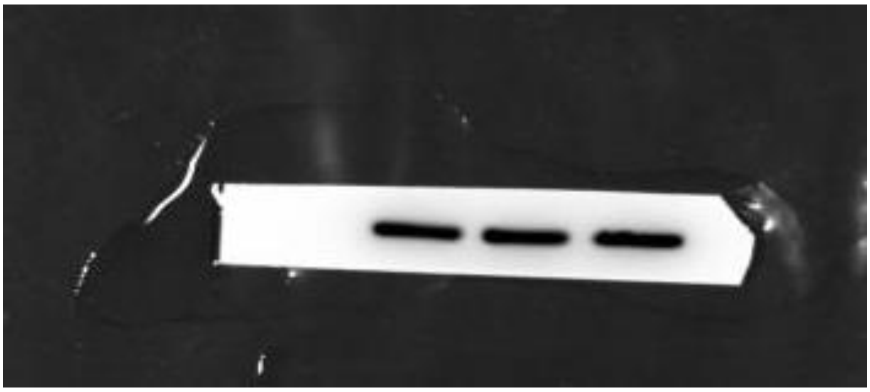

Original strip of GAPDH in Fig.B
